# Frailty-Informed Assessment in Peritoneal Dialysis: A Multicenter Survey of Current Practice and Implementation Barriers in Spain

**DOI:** 10.3390/geriatrics11050140

**Published:** 2026-10-06

**Authors:** Estefanya García-Menéndez, Jose Portolés, Luisa Sánchez-García, Jesús Delgado, Paula Jara Caro Espada, Yanieli Hernández, Ana Sánchez Horrillo, Almudena Vega, R. Haridian Sosa Barrios, María José Fernández-Reyes, Nerea Begoña Boldova, Fernando Tornero, Karen Rivero, Victoria Oviedo, Marina González de Rivera, Ana Tato, Laura Espinel, Laura Cueto, Laura Rodríguez-Osorio, Mayra Ortega, Paula López-Sánchez

**Affiliations:** 1 Nephrology Department, Instituto de Investigación Puerta de Hierro-Segovia de Arana(IDIPHISA), Hospital Universitario Puerta de Hierro-Majadahonda, 28222 Madrid, Spain; estefanialisset.garcia@salud.madrid.org (E.G.-M.); nefro_metodologia@yahoo.com (P.L.-S.); 2 Medicine Department, Facultad de Medicina, Universidad Autónoma de Madrid, 28029 Madrid, Spain; 3 Department of Nephrology, Hospital Universitario Rio Hortega, 47012 Valladolid, Spain; 4 Department of Nephrology, Hospital Universitario Clinico San Carlos de Madrid, 28040 Madrid, Spain; 5 Department of Nephrology, Hospital Universitario Doce de Octubre, 28041 Madrid, Spain; 6 Department of Nephrology, Hospital Universitario La Paz, 28046 Madrid, Spain; 7 Department of Nephrology, Hospital Universitario de la Princesa, ISS-Princesa, 28006 Madrid, Spain; 8 Department of Nephrology, Hospital Universitario Gregorio Marañon, 28007 Madrid, Spain; 9 Department of Nephrology, Hospital Universitario Ramón y Cajal, 28034 Madrid, Spain; 10 Department of Nephrology, Hospital Universitario General de Segovia, 40002 Segovia, Spain; 11 Department of Nephrology, Hospital Universitario Guadalajara, 19002 Guadalajara, Spain; 12 Department of Nephrology, Hospital Universitario del Sureste, 28500 Madrid, Spain; 13 Department of Nephrology, Hospital Universitario de Salamanca, 37007 Salamanca, Spain; 14 Department of Nephrology, Hospital Universitario Rio Carrión, 34004 Palencia, Spain; 15 Department of Nephrology, Hospital Universitario Fundación Jiménez Diaz, 28040 Madrid, Spain; 16 Department of Nephrology, Hospital Universitario Fundación Alcorcon, 28922 Madrid, Spain; 17 Department of Nephrology, Hospital Universitario Getafe, 28905 Madrid, Spain; 18 Department of Nephrology, Complejo Hospital Universitario Toledo, 45004 Toledo, Spain; 19 Department of Nephrology, Hospital Universitario Villalba, 28400 Madrid, Spain; 20 Department of Nephrology, Hospital Universitario Infanta Leonor, 28031 Madrid, Spain

**Keywords:** frailty, peritoneal dialysis, sarcopenia, nutritional assessment, quality of life, comprehensive assessment, bioimpedance, muscle ultrasound, patient-centered care, chronic kidney disease

## Abstract

**Background/Objectives:** Frailty is increasingly recognized as a major determinant of adverse outcomes in patients receiving peritoneal dialysis (PD), yet it is not routinely incorporated into clinical practice. Because PD relies heavily on patient autonomy and self-management, frailty, sarcopenia, malnutrition, and impaired quality of life may compromise both outcomes and technique retention. This study aimed to describe current clinical practices related to frailty-informed assessment in PD units participating in the GCDP network and to identify barriers to implementing a more comprehensive, patient-centered approach. **Methods:** We conducted a descriptive, observational, cross-sectional study based on a survey distributed to PD units belonging to the Grupo Centro de Diálisis Peritoneal (GCDP), a collaborative network of 24 hospitals across four autonomous communities in Spain. Physicians and nurses completed the questionnaire between January and February 2025. The survey explored seven domains: use of frailty scales, nutritional screening tools, dynamometry, muscle ultrasound, bioimpedance, quality of life instruments, and digital applications for patient follow-up. **Results:** Twenty of 24 centers completed the survey. Although all units considered frailty clinically relevant, only 11 assessed it at PD initiation and only 5 performed periodic or systematic re-evaluation. Nutritional screening tools were used in 13 centers, most commonly the Malnutrition Inflammation Score. Twelve centers had a dynamometer, though its use was not standardized. All centers used bioimpedance—primarily for hydration assessment—while muscle ultrasound was rarely applied in scheduled follow-up. Only 7 centers assessed quality of life. The main reported barriers were lack of time in routine practice and the absence of specific guidelines and training. **Conclusions:** Frailty-related assessment across participating GCDP units remains heterogeneous and incompletely implemented despite broad recognition of its clinical relevance. The identified gaps in standardized protocols, training, and integration of assessment tools highlight priorities for future implementation research aimed at incorporating frailty-informed, patient-centered assessment into routine PD care.

## 1. Introduction

Frailty is a multidimensional clinical syndrome characterized by a progressive decline in physiological reserve, reduced resilience to stressors, and heightened vulnerability to adverse outcomes [1]. Although traditionally associated with advanced age, frailty is increasingly recognized among individuals living with chronic kidney disease (CKD), where its prevalence is substantially higher than in community-dwelling older adults. In dialysis populations, frailty has been consistently linked to increased mortality, higher hospitalization rates, functional decline, loss of independence, and impaired quality of life [2,3]. As the global dialysis population continues to age, the clinical relevance of frailty is becoming even more pronounced [4].

Peritoneal dialysis (PD) offers several advantages for older or comorbid individuals—greater treatment autonomy, preservation of residual kidney function, and improved flexibility for patients and caregivers. However, PD also places considerable demands on patient self-management. Tasks such as aseptic technique, cycler operation, and problem recognition require adequate physical, cognitive, and functional capacity [5]. Frailty, sarcopenia, malnutrition, and dependency therefore represent key determinants of PD feasibility, technique survival [6], poorer outcomes after kidney transplantation [7], and overall patient experience and independence [8]. A multicenter study showed that PD in patients older than 65 years is a valid treatment option, with similar technique survival, although with a higher risk of peritonitis. Unfortunately, frailty scales were not available to estimate their effect on adverse events and complications [9].

International societies, including the International Society for Peritoneal Dialysis (ISPD), increasingly advocate for the integration of structured frailty assessment and comprehensive geriatric evaluation into the routine management of PD patients [10]. Frailty prevalence among patients receiving PD has been reported to range from 34.2% to 69.4% in individual cohorts [11,12], while a prevalence of 75% has been reported among older patients initiating dialysis [13]. Frailty has also been associated with an increased risk of hospitalization and mortality [11,14]. Despite the growing evidence base and availability of validated tools, the actual adoption of frailty-informed practices remains inconsistent across countries and PD units [15]. Implementation barriers include limited training, absence of standardized protocols, and time constraints in routine care. As a result, frailty often remains under-recognized and insufficiently addressed, even though it has major implications for modality selection, individualized goal-directed treatment, symptom management, and shared decision-making.

Spain provides a particularly relevant setting in which to explore these issues. Kidney replacement therapy in Spain is provided within a universal, publicly funded healthcare system. Hemodialysis (HD), PD, and home-HD are available, with modality choice based on informed patient preference whenever more than one option is clinically appropriate; eligibility is determined by comprehensive clinical assessment rather than age, all treatment-related costs are fully covered by the National Health System and PD is performed by the patient or a trained family caregiver. The Spanish PD population is aging rapidly, and PD units differ markedly in experience, staffing models, and available resources. The Central Peritoneal Dialysis Group (Grupo Centro de Diálisis Peritoneal, GCDP)—a multicenter collaborative network of 24 hospitals across four regional health systems serving more than 12 million people in Spain—offers a unique opportunity to examine real-world practice patterns across diverse clinical environments [9].

Recently, Jassal and van Loon raised two important questions: can frailty assessment be integrated into routine clinical practice, and, if so, does it provide additional value to patient care? [16]. Frailty, sarcopenia, nutritional impairment, functional dependency, and HRQoL represent distinct but frequently overlapping clinical constructs. These domains might be particularly relevant in patients receiving PD, in whom age-related physiological decline coexists with kidney failure and treatment-specific factors. Importantly, vulnerability in patients receiving dialysis is not solely determined by chronological age. Kidney failure has been increasingly recognized as a state of premature or accelerated biological aging, characterized by chronic inflammation, oxidative stress, cellular senescence, and loss of physiological reserve [17]. Consequently, aging-related syndromes such as frailty and sarcopenia might also occur in younger patients with kidney failure; indeed, a substantial burden of frailty has been reported in dialysis patients younger than 65 years [18]. In patients receiving PD, these age- and kidney disease-related changes coexist with treatment-specific factors, including continuous glucose exposure, peritoneal protein losses, and alterations in fluid status, which may further contribute to nutritional and body-composition changes [19,20]. At the same time, PD is a complex, largely self-administered treatment, and frailty may compromise the ability to remain on the technique [21]. In this study, these domains were considered separately as complementary components of a broader frailty-informed, patient-centered assessment rather than as interchangeable measures of frailty.

The present study aims to describe how frailty-related assessments and complementary domains, including nutritional status, muscle strength, body composition, and HRQoL are incorporated into routine care in PD units participating in the GCDP network, and to identify perceived barriers to their implementation. By characterizing current practice and unmet needs within this regional network, we seek to highlight critical gaps and inform the development of standardized, patient-centered PD care pathways.

We hypothesized that the systematic assessment of frailty and complementary multidimensional domains remains heterogeneously implemented across participating PD units.

## 2. Materials and Methods

### 2.1. Study Design and Setting

We conducted a descriptive, observational, cross-sectional study aimed at characterizing current practices related to frailty, nutritional status, sarcopenia assessment, and patient-reported outcomes in Spanish peritoneal dialysis (PD) units within the GCDP network. All participating centers were public hospitals within the Spanish National Health System. The GCDP meets twice yearly and promotes standardized PD management, quality benchmarking, and clinical research across participating centers. The study was reported in accordance with the Consensus-Based Checklist for Reporting of Survey Studies (CROSS), provided as Supplementary Material S1 [22].

### 2.2. Survey Development and Content

A structured questionnaire was designed by senior nephrologists within the GCDP to describe center-level organizational characteristics and routine clinical practices. In the absence of validated questionnaires, items were selected to explore clinically relevant domains related to frailty-informed assessment in PD care. It was reviewed by another three nephrologists to reach consensus on its content, clinical relevance, and clarity before distribution to the remaining participating centers. Therefore, no formal validation or pilot testing was performed. The instrument consisted of two sections:

1. General unit characteristics
  ○ Number of prevalent and incident PD patients;
  ○ Staffing structure;
  ○ Availability of technical resources.
2. Assessment practices across seven predefined complementary domains relevant to multidimensional, frailty-informed PD care:
  ○ Use of frailty scales;
  ○ Use of nutritional screening tools;
  ○ Availability and application of handgrip dynamometry;
  ○ Use of muscle ultrasound for body composition assessment;
  ○ Use of bioimpedance analysis;
  ○ Administration of health-related quality of life questionnaires;
  ○ Use of digital applications for structured patient follow-up.

Most items were closed-ended or multiple choice to ensure consistency of responses across centers. The complete questionnaire and the study flow diagram are provided in Supplementary Materials S2 and Figure S1.

### 2.3. Participants and Data Collection

The questionnaire was distributed by email to all GCDP centers (*n* = 24) and was completed jointly by the nephrologist responsible for the PD unit and the PD nurse. Each participating center submitted a single consolidated questionnaire and respondents were instructed to report actual routine clinical practice within their PD unit and not their individual opinions. Data were collected between January and February 2025. No patient-level information was obtained.

### 2.4. Data Management and Analysis

Survey responses were anonymized at the center level and compiled for descriptive statistical analysis, which was carried out by other staff. Given the exploratory nature of the study, only descriptive analyses were performed. Results are presented as absolute frequencies, percentages, and medians with interquartile ranges where relevant. No inferential statistical comparisons were planned. Data were analyzed using Microsoft Excel.

### 2.5. Ethical Considerations

This study collected exclusively center-level information on organizational and clinical practices and did not involve patient-level or identifiable personal data. The study was reviewed by the Research Ethics Committee of Hospital Universitario Puerta de Hierro Majadahonda, which classified it as research aimed at evaluating healthcare practice and quality of care and determined that formal ethical approval was not required (CEIm reference EXE-20/26).

## 3. Results

### 3.1. General Characteristics of Participating Units

A total of 20 out of 24 PD units belonging to the GCDP completed the survey, representing centers across four Spanish regional health systems. All participating centers completed all survey items, with no item-level missing data. Participating units reported a median of 24 prevalent PD patients (interquartile range [IQR] 15–34) and 10 incident patients per year (IQR 6–19). Nineteen centers treated more than 20 individual PD patients over the course of the year. Regarding program experience, 2 units had <10 years of activity, 4 had 10–15 years, and the remainder had longer-standing PD programs.

Staffing models showed a median of 1 nephrologist (IQR 1–1.5) and 2 nurses (IQR 1.5–2) dedicated to PD care per unit.

### 3.2. Frailty Assessment

All responding centers recognized frailty as a clinically relevant component of comprehensive PD assessment. However, only 11 units reported evaluating frailty at the time of PD initiation, and just 5 conducted periodic or systematic follow-up.

The FRAIL scale was the most frequently used frailty screening tool (8 centers). Functional capacity tests were employed in only 2 units.

Nutritional Assessment: Nutritional evaluation practices varied considerably. Thirteen units incorporated formal nutritional screening tools into routine care, most commonly the Malnutrition Inflammation Score (MIS). Only four centers reported access to a dedicated nutritionist, with follow-up intervals ranging from biannual assessment to evaluation only in selected cases.

### 3.3. Sarcopenia and Muscle Assessment

Integrated assessment of sarcopenia—including both muscle mass and muscle function—was performed regularly in only three PD units.

Although 12 centers had access to a handgrip dynamometer, its use was inconsistent and was not standardized. All centers had bioimpedance analysis available and used it routinely; however, assessments focused primarily on hydration status. Only two centers applied validated equations to estimate appendicular skeletal muscle mass.

**Figure 1:**
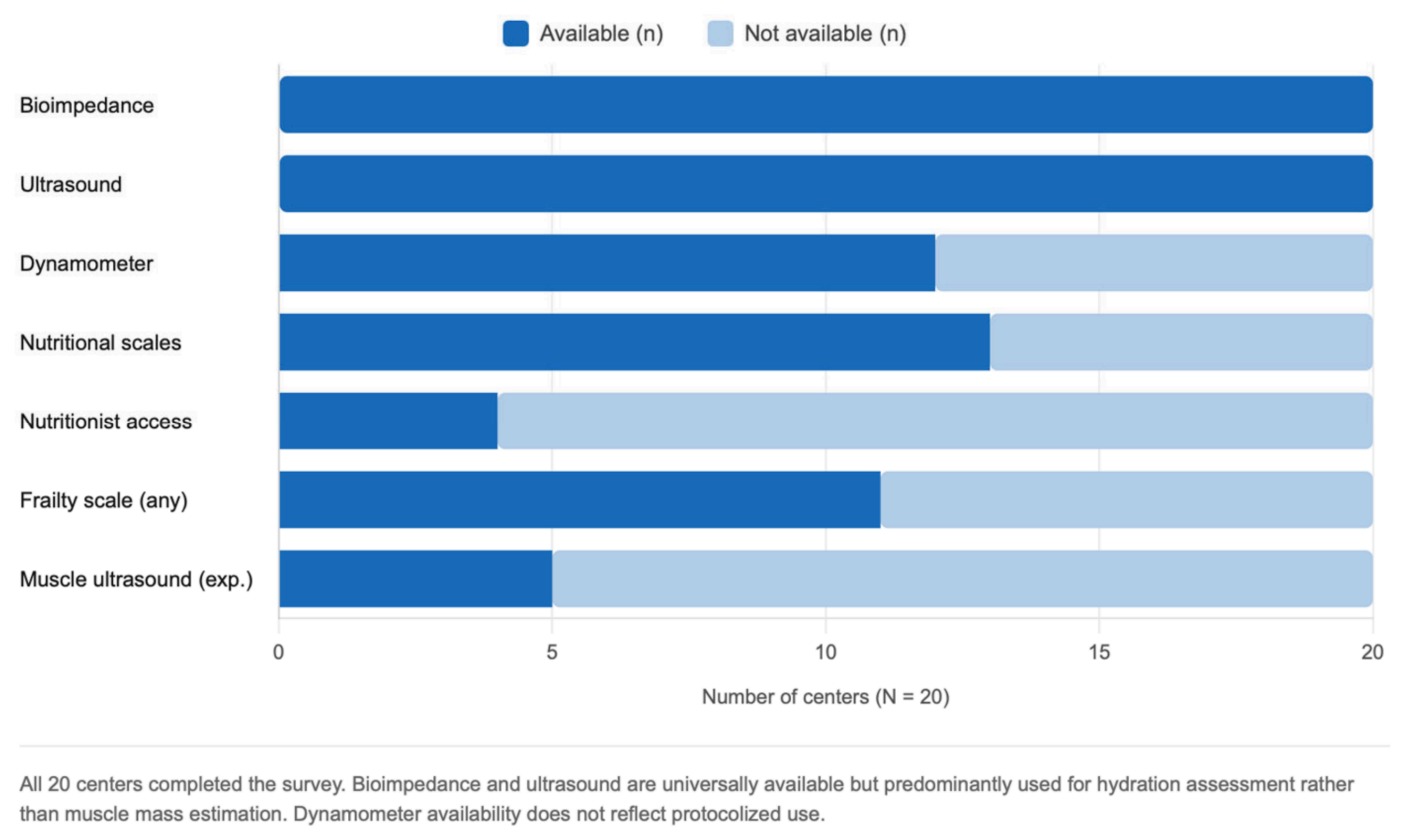
Availability of tools or resources in peritoneal dialysis units for the assessment of frailty, sarcopenia, and nutrition.

Ultrasound equipment was universally available at the hospital level, yet its use for muscle assessment was rare. Five PD units reported staff capability to perform muscle ultrasound, but only one conducted scheduled semiannual evaluations of muscle mass (Figure 1).

### 3.4. Quality-of-Life Assessment

**Figure 2:**
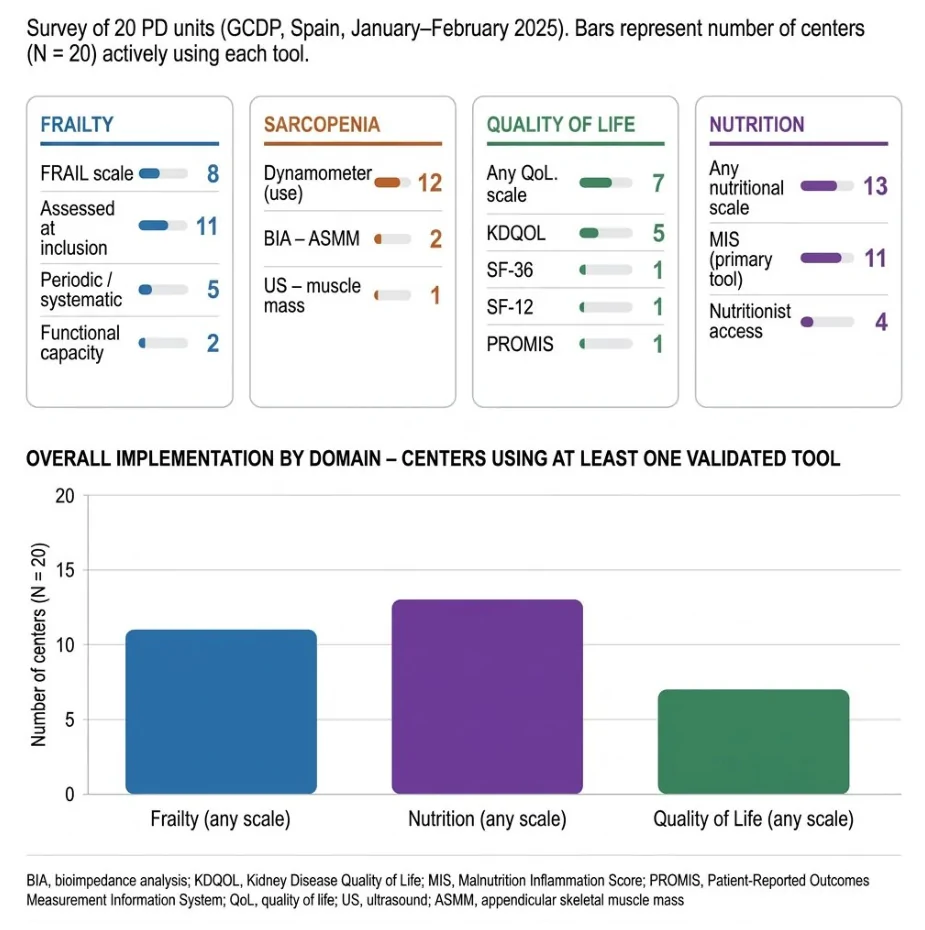
Use of different tools for frailty, sarcopenia, nutritional, and health-related quality-of-life assessment across participating peritoneal dialysis units.

While all units recognized the importance of measuring health-related quality of life (HRQoL), only seven centers incorporated HRQoL tools into clinical practice. Five used the Kidney Disease Quality of Life instrument (KDQOL), one used the SF-36, and one used the SF-12. A single center reported experience with the Patient-Reported Outcomes Measurement Information System (PROMIS), aligned with the recent Spanish ICHOM PROMIS adaptation [23] (Figure 2).

### 3.5. Digital Tools and Follow-Up Applications

All PD units reported using some form of structured electronic medical record tailored to PD care. These systems came from four different providers and varied widely in their functionality and integration. None of the platforms included a comprehensive set of fields enabling standardized monitoring of frailty, nutritional status, HRQoL, and dependency measures.

### 3.6. Perceived Barriers and Training Needs

The most frequently reported barrier to implementing frailty-informed and comprehensive assessment protocols was lack of time in routine clinical practice (9 centers). Additional barriers included the absence of specific clinical guidelines for assessment and intervention (5 centers).

All unit leads indicated that they had not received formal training in the past 5 years on frailty, sarcopenia, or HRQoL evaluation. Despite broad agreement that frailty influences modality choice and may also affect patient prognosis, 20% of respondents reported uncertainty about how incorporating these assessments would modify their day-to-day clinical workflow.

## 4. Discussion

This study provides a multicenter overview of how frailty-related assessments and complementary multidimensional domains are incorporated into routine practice across GCDP-participating PD units. Despite widespread recognition of the clinical importance of frailty, our findings reveal substantial gaps not only in the implementation of structured frailty assessment tools, but also in nutritional evaluation, sarcopenia screening, and patient-reported outcome measures. All participating centers were public hospitals operating within the Spanish National Health System, precluding comparisons according to public versus private funding models. Nevertheless, participating PD programs differed in size, years of experience, and staffing. This heterogeneity suggests that differences in the implementation of multidimensional assessment might reflect not only resource availability but also local organization, staff training, and institutional priorities. These results highlight the need to develop standardized protocols and strengthen training to improve the quality and consistency of multidimensional patient-centered assessment in PD programs.

The low uptake of frailty screening tools is particularly notable. Although multiple instruments have been validated in geriatric and nephrology populations and are highly feasible for routine use, their application remains limited in PD units. Systematic assessment enables early identification of patients at risk of adverse outcomes and facilitates personalized intervention planning [15]. However, as reflected in our survey, barriers such as lack of training, insufficient time in routine workflows, and absence of unified guidelines continue to hinder implementation. These challenges are consistent with international experience. In a large Italian survey of 337 nephrologists, most clinicians perceived CKD as a geriatric condition but demonstrated limited familiarity with geriatric tools [24]. Similarly, Nixon and colleagues showed that implementing a frailty screening program followed by geriatric assessment required targeted training and uncovered significant unmet needs among both dialysis and non-dialysis CKD patients, with dialysis patients exhibiting greater vulnerability [25].

Our results also reflect broader uncertainties regarding the definition and operationalization of frailty in CKD. There is still no consensus on the most appropriate frailty construct for renal populations. A recent Spanish study in HD patients showed that older individuals exhibited the highest degrees of frailty, dependency, and mobility impairment, reinforcing the need for systematic assessment and structured intervention pathways to mitigate these complications [26]. Yet frailty is not solely an age-related condition; it may emerge at any stage of chronic illness and independently predict mortality regardless of age or disease severity [27]. Kidney failure has been associated with features of accelerated biological aging [17,28], and frailty is also highly prevalent among younger patients receiving dialysis [18]. Thus, the coexistence of frailty, nutritional impairment, sarcopenia, and functional decline in the dialysis population may reflect the combined effects of biological aging, kidney failure, multimorbidity, and dialysis-related factors rather than chronological age alone. This reinforces the rationale for assessing these related but distinct domains across the PD population.

The observed implementation gaps are particularly relevant in the context of current recommendations promoting individualized, goal-directed PD care that incorporates patient priorities, functional status, and HRQoL alongside conventional dialysis parameters [16,29,30].

Nutritional assessment represents an additional domain where practice gaps were evident. This is particularly relevant in PD, where nutritional impairment and PEW are common and associated with adverse outcomes [31,32,33,34,35]. Although peritoneal protein losses are well documented, their relationship with nutritional status and clinical outcomes remains complex [36,37,38]. Nutritional assessment should therefore complement, rather than substitute for, frailty evaluation, as these represent distinct but frequently overlapping clinical constructs.

Sarcopenia is defined by low muscle mass and strength and is associated with increased morbidity, hospitalization, and frailty [39]. Sarcopenia is a distinct condition that frequently overlaps with frailty but should not be considered synonymous with it. Although multiple tools exist for its assessment—including bioimpedance, handgrip strength, and, more recently, muscle ultrasound—no gold standard has been established. Our findings show that despite broad availability of dynamometers and ultrasound equipment, their routine use remains limited, likely due to insufficient training and lack of standardized protocols. This is concerning because handgrip strength is an accessible, inexpensive, and reliable predictor of adverse outcomes in CKD [40,41,42].

Health-related quality of life (HRQoL) is increasingly recognized as a key determinant in modality selection and long-term PD retention. Modern PD guidelines advocate for goal-directed dialysis, emphasizing alignment of treatment with patient-defined priorities [10]. PD confers advantages in lifestyle preservation, dietary flexibility, employment, family support, and reduced hospital exposure. Despite this, our survey shows that HRQoL measures are rarely used systematically. PROMIS tools, increasingly promoted by national nephrology societies [23] and validated across kidney populations [43,44], remain underutilized.

Evidence on the implementation of frailty-informed assessment in Spanish PD practice remains limited. Previous studies have primarily addressed the prevalence and prognostic implications of frailty [21,45,46], whereas our findings highlight a complementary issue: the limited and heterogeneous integration of structured assessment into routine clinical workflows. Broader studies will be needed to determine whether these findings extend beyond the GCDP network.

Training of staff, integration of validated tools into electronic systems, and investment in dedicated resources are essential. Moving beyond numerical adequacy targets toward individualized, person-centered care aligns with modern nephrology principles and is supported by scientific societies [10].

From an implementation perspective, our findings identify three potentially modifiable barriers: limited staff training, insufficient time within routine workflows, and the absence of standardized assessment pathways. Future implementation studies should therefore evaluate whether targeted training, simplified assessment protocols, and integration of validated tools into electronic clinical systems can improve the feasibility and consistency of frailty-informed assessment in routine PD care.

**Figure 3:**
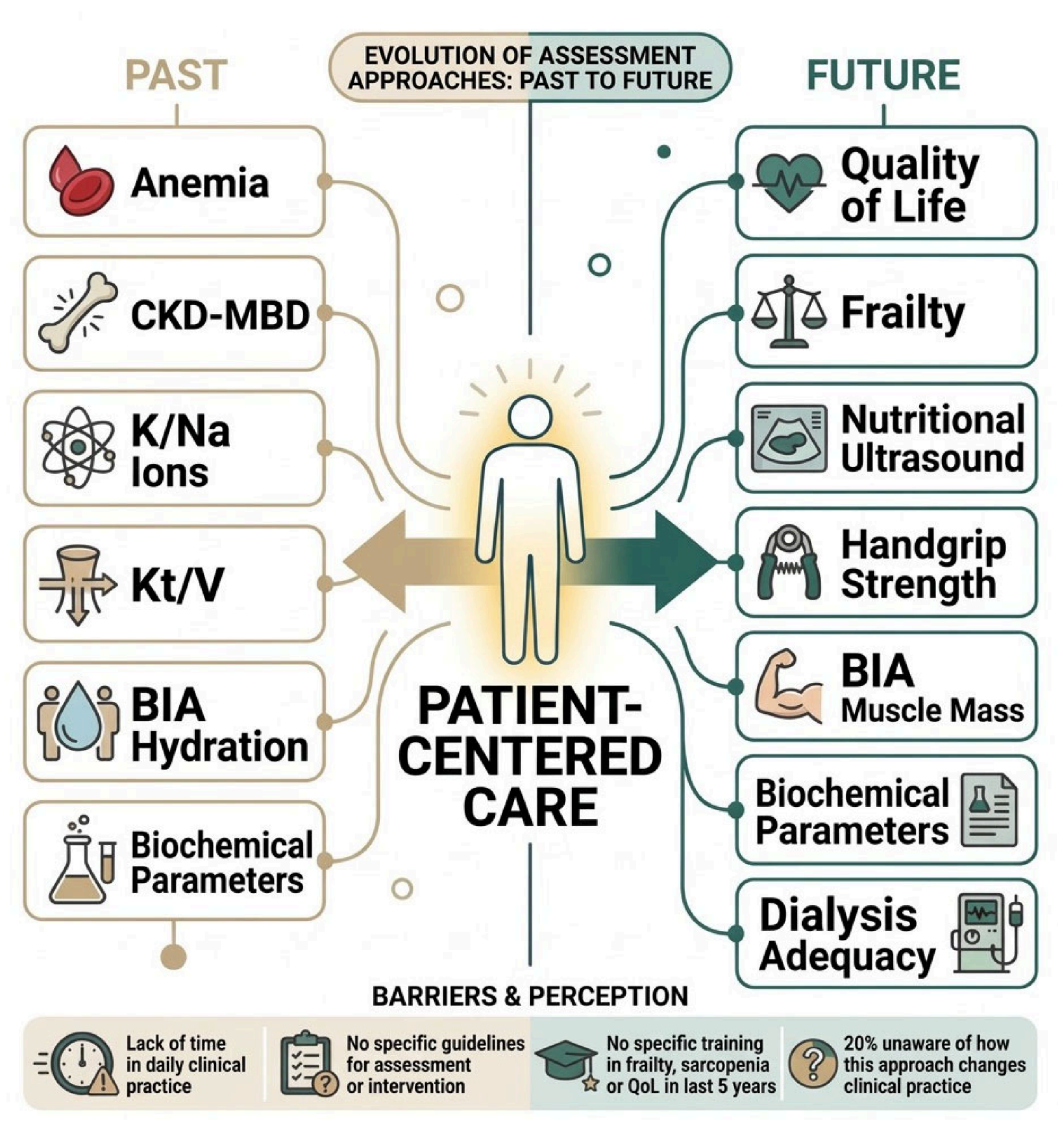
Proposed conceptual framework for multidimensional, frailty-informed, patient-centered assessment in peritoneal dialysis units.

Based on our survey findings, we propose a practical framework for integrating frailty-informed assessment into routine PD care (Figure 3). This model complements conventional dialysis monitoring by incorporating frailty, nutritional status, muscle strength, body composition, and health-related quality of life. The proposed framework draws on the multidimensional principles of comprehensive geriatric assessment [47]. Although not formally validated, this conceptual framework is intended to generate hypotheses and guide future implementation studies rather than represent an evidence-based clinical pathway.

Our study has several limitations. First, the survey included 20 PD units from the GCDP network and, therefore, the findings should not be considered representative of all PD units in Spain. Although the response rate was high, nonresponse bias cannot be excluded. Second, data were self-reported at the center level, with each center providing a single consolidated response, and intended to reflect routine unit-level practice rather than individual professional practice. Nevertheless, informant and reporting bias remain possible, and the survey did not formally distinguish between institutionally approved protocols and locally adopted routine practices. Third, the questionnaire was specifically developed for this descriptive study and, although its content relevance and item clarity were reviewed by three experienced GCDP nephrologists, it did not undergo formal pilot testing or psychometric validation, which may limit the reliability and reproducibility of the findings. Moreover, the survey addressed selected domains of frailty-informed assessment rather than comprehensive geriatric assessment and did not evaluate cognition, falls, polypharmacy, activities of daily living, or caregiver and social factors. Finally, the cross-sectional design and absence of patient-level data preclude assessment of associations between these practices and clinical outcomes. Nevertheless, the multicenter design, high participation rate, and diversity of participating PD programs provide a valuable real-world overview of current practices within the GCDP network and identify priorities for future implementation studies.

## 5. Conclusions

Frailty is a frequent and clinically meaningful condition in PD patients. Our multicenter survey demonstrates substantial heterogeneity in how frailty and related domains—nutritional status, sarcopenia, functional capacity, and quality of life—are assessed across GCDP-participating PD units. Despite the availability of validated tools, their use is inconsistent and largely unstandardized. Understanding current practices is essential to identify barriers and guide the development of context-adapted strategies. Implementing clear guidelines, strengthening staff training, and allocating dedicated time for multidimensional frailty-informed assessment may facilitate its integration into routine PD care. Whether these strategies translate into improved clinical outcomes or HRQoL should be evaluated in prospective implementation studies.

## Data Availability

The data presented in this study are available on request from the corresponding author due to privacy/ethical restrictions.

## Supplementary Materials

The following supporting information can be downloaded at: https://www.mdpi.com/article/10.3390/geriatrics11050140/s1. Supplementary Material S1. CROSS Checklist; Supplementary Material S2. Questionnaire on Current Assessment Practices in Peritoneal Dialysis Units; Supplementary Figure S1. Study flow diagram.

## Abbreviations

The following abbreviations are used in this manuscript:

ASMM	Appendicular Skeletal Muscle Mass
BIA	Bioimpedance Analysis
CKD	Chronic Kidney Disease
CROSS	Consensus-Based Checklist for Reporting of Survey Studies
FRAIL	Fatigue, Resistance, Ambulation, Illnesses, and Loss of Weight
GCDP	Grupo Centro de Diálisis Peritoneal
HD	Hemodialysis
HRQoL	Health-Related Quality of Life
ICHOM	International Consortium for Health Outcomes Measurement
IQR	Interquartile Range
ISPD	International Society for Peritoneal Dialysis
KDQOL	Kidney Disease Quality of Life
Kt/V	Dialysis adequacy index: urea clearance (K) × treatment time (t) divided by the volume of distribution of urea (V)
MIS	Malnutrition–Inflammation Score
PD	Peritoneal Dialysis
PEW	Protein–Energy Wasting
PROMIS	Patient-Reported Outcomes Measurement Information System
PROMs	Patient-Reported Outcome Measures
SF-12	12-Item Short Form Health Survey
SF-36	36-Item Short Form Health Survey
