# Supplementary material for: Frailty-Informed Assessment in Peritoneal Dialysis: A Multicenter Survey of Current Practice and Implementation Barriers in Spain
Source: Geriatrics (Basel). 2026 Oct 6;11(5):140. doi: 10.3390/geriatrics11050140 (PMC13704586; doi:10.3390/geriatrics11050140)
Supplement: Supplementary file 1 [file geriatrics-11-00140-s001.zip › geriatrics-4470639-supplementary.pdf]

Supplementary Material S1. CROSS Checklist

Consensus-Based Checklist for Reporting of Survey Studies (CROSS)

Study: Frailty-Informed Assessment in Peritoneal Dialysis: A Multicenter Survey of Current Practice and Implementation Barriers in Spain

Section	Item	CROSS requirement (concise)	Status / location in revised manuscript
Title & Abstract	**1a**	Identify the work as a survey and state the design in the title or abstract.	MET — Revised title includes “Multicenter Survey”; Abstract/Methods identify the cross-sectional design.
Title & Abstract	**1b**	Give an informative abstract covering rationale, objective, methods, main results, interpretation and conclusion.	MET — Abstract.
Introduction	**2**	Explain background, prior evidence, rationale and why the survey was needed.	MET — Introduction.
Introduction	**3**	State the specific aim/objective (and hypothesis where relevant).	MET — End of Introduction; explicit hypothesis added.
Methods	**4**	State the study design using a standard term.	MET — Multicenter, observational, cross-sectional survey study.
Methods – Data collection	**5a**	Describe questionnaire structure, number of items/sections, formats and domains.	MET / FINAL NUMBER TO VERIFY — Survey Development and Content; full questionnaire in Supplementary Material S1.
Methods – Data collection	**5b**	Describe instruments used to measure specific concepts and provide appropriate measurement information/references.	PARTLY APPLICABLE — FRAIL, MIS, SPPB, KDQOL, SF-36/SF-12, PROMIS, etc. were not administered by investigators; the survey recorded routine use by units. Clarify and cite key instruments where discussed.
Methods – Data collection	**5c**	Describe questionnaire pretesting/piloting if performed.	MET — Reviewed/discussed by three experienced GCDP nephrologists for content, clinical relevance and clarity; no formal pilot testing.
Methods – Data collection	**5d**	Provide the complete questionnaire when possible.	MET — Supplementary Material S2
Methods – Sample	**6a**	Describe study population, setting, locations and eligibility criteria.	MET — All 24 GCDP PD units across four Spanish regional health systems were eligible/invited; participating centers were public hospitals in the Spanish National Health System.
Methods – Sample	**6b**	Describe the sampling approach and relevant locations.	MET — Census of the GCDP network: all 24 member units invited; no subset sampled.
Methods – Sample	**6c**	Report sample size and any sample-size calculation.	MET— All 24 eligible units invited; 20 participated. No formal sample-size calculation required for this network-wide descriptive survey.
Methods – Sample	**6d**	Describe representativeness of the achieved sample.	MET — 20/24 units (83.3%); Limitations state findings are not representative of all PD units in Spain, pero sí del entorno GCDP.
Methods – Administration	**7a**	Describe survey administration, contact mode and respondent pathway.	MET — Excel questionnaire emailed to the nephrologist responsible for / PD lead at each of 24 GCDP units; one center-level response per unit.
Methods – Administration	**7b**	Report the survey/data-collection time frame.	MET — January to February 2025.
Methods – Administration	**7c**	For non-web surveys, describe procedures used to minimize data-entry error.	MET — Returned Excel files were compiled into a centralized database. If there were any doubts about the response, we contacted the center.
Methods – Preparation	**8**	Describe preparation before launch, if applicable.	MET / N/A — No interviewer training/advertising required; questionnaire reviewed by three GCDP nephrologists before distribution.
Methods – Ethics	**9a**	Report ethical review/approval and consent considerations as applicable.	MET — CEIm Hospital Universitario Puerta de Hierro Majadahonda, EXE-20/26; formal ethical approval not required. No patient-level data; patient consent not applicable.
Methods – Ethics	**9b**	Describe anonymity/confidentiality and safeguards against unauthorized access.	MET — Center-level data and no patient-identifiable data stated. The staff evaluating the survey did not have access to data from the center
Methods – Statistics	**10a**	Describe statistical methods/analytical approach and software.	MET — n, %, median and IQR; no inferential testing.
Methods – Statistics	**10b**	Report modifications/transformations of variables used in analysis.	N/A
Methods – Statistics	**10c**	Describe missing-data handling and item-level missingness.	MET — All 20 participating centers completed all items; no item-level missing data; no imputation required.
Methods – Statistics	**10d**	State how potential nonresponse error/bias was addressed.	MET — 20/24 response (83.3%); four did not participate; potential nonresponse bias acknowledged.
Methods – Statistics	**10e**	For longitudinal surveys, describe loss-to-follow-up handling.	N/A — Cross-sectional survey.
Methods – Statistics	**10f**	State whether weighting/propensity adjustment for nonrepresentativeness was used.	N/A — No weighting/propensity adjustment.
Methods – Statistics	**10g**	Describe sensitivity analyses, if performed.	N/A — No sensitivity analyses; descriptive study.
Results – Respondents	**11a**	Report numbers at each study stage; consider a flow diagram.	MET — 24 invited; 20 participated; 4 did not participate; Supplementary Figure S2
Results – Respondents	**11b**	Give reasons for nonparticipation when known.	PARTLY APPLICABLE — Four units did not participate. Do not assign reasons;
Results – Respondents	**11c**	Report response rate and its calculation/definition.	MET — 83.3% = 20 completed questionnaires / 24 invited units.

Section	Item	CROSS requirement (concise)	Status / location in revised manuscript
Results – Respondents	**11d**	For web surveys, explain unique-visitor and web participation/completion metrics.	N/A — Non-web: Excel questionnaire sent directly to one designated PD lead per center.
Results – Descriptive	**12**	Describe respondent/unit characteristics and assessed variables/outcomes.	MET — Results/Table 1: center size, experience, staffing and assessment practices.
Results – Main findings	**13a**	Report principal estimates; add CIs/p values when inferential estimates apply.	MET AS APPLICABLE — Frequencies/percentages and medians/IQRs reported; inferential estimates not applicable.
Results – Main findings	**13b**	For multivariable models, report model building, fit and assumptions.	N/A — No multivariable analysis.
Results – Main findings	**13c**	Report sensitivity analyses and missing-data sensitivity analyses when relevant.	N/A — No sensitivity analysis; no item-level missing data.
Discussion	**14**	Discuss limitations, including bias, imprecision and nonrepresentativeness.	MET — Expanded Limitations: regional frame, 20/24 participation, nonresponse, single center-level respondent, informant/reporting bias, no formal pilot/psychometric validation, cross-sectional design, no patient-level outcomes.
Discussion	**15**	Interpret findings cautiously and identify future research needs.	MET — Causal/outcome claims softened; Figure 3 hypothesis-generating; prospective implementation studies proposed.
Discussion	**16**	Discuss external validity/generalizability.	MET — Explicitly not representative of all Spanish PD units.
Other	**17**	State funding and whether funders influenced design, conduct, analysis or reporting.	MET —funder no role in design, implementation, analysis, interpretation and manuscript preparation.
Other	**18**	Declare potential conflicts of interest.	MET— Disclosure includes lecture honoraria from PERSAN and congress support from PALEX; all authors' disclosures complete.
Other	**19**	Acknowledge contributors/organizations and their contribution where appropriate.	MET— Check

Reference: Sharma A, Minh Duc NT, Luu Lam Thang T, et al. A Consensus-Based Checklist for Reporting of Survey Studies (CROSS). J Gen Intern Med. 2021;36(10):3179–3187. doi:10.1007/s11606-021-06737-1.

Before submission, verify: final questionnaire item count; actual data-entry verification procedure; confidentiality/data-storage safeguards; statistical software/version; reasons for nonparticipation only if documented; and explicit role of funding source(s).

Supplementary Material S2

Questionnaire on Current Assessment Practices in Peritoneal Dialysis Units

Section 1. General characteristics of the PD unit

1.- How many prevalent patients are currently receiving PD in your unit?

2 .- How many incident PD patients were treated in your unit during the previous year?

3.- How many nephrologists are dedicated to the PD program?

4.- How many nurses are dedicated to the PD program?

5.- How many years has your PD program been active?

Section 2. Frailty assessment

6. Do you routinely assess frailty in patients receiving PD?
7. Which frailty assessment tool(s) do you use?
    - FRAIL scale
    - Short Physical Performance Battery (SPPB)
    - Other (please specify)
8. Is frailty assessed at PD initiation?
9. Is frailty reassessed during follow-up? If yes, at what interval?
10. Does frailty assessment influence PD prescription or treatment decisions?
11. If you do not routinely assess frailty, what are the main reasons?

- Lack of time
- Lack of specific training
- Lack of guidelines/protocols
- I do not consider frailty assessment useful
- Other (please specify)

Section 3. Nutritional assessment

12. Do you routinely use a nutritional assessment tool?
13. Which tool(s) do you use?

- Malnutrition–Inflammation Score (MIS)
- Subjective Global Assessment (SGA)
- Other (please specify)

14. Is a dietitian/nutritionist available for PD patients?
15. If yes, how frequently are patients assessed by the dietitian/nutritionist?
16. If nutritional assessment tools are not routinely used, what are the main reasons?

- Lack of time
- Lack of training
- Not considered useful
- Other (please specify)

Section 4. Sarcopenia and muscle assessment

17. Do you routinely screen for sarcopenia?
18. Is sarcopenia assessed at PD initiation?
19. Is sarcopenia reassessed during follow-up? If yes, at what interval?
20. Does sarcopenia assessment influence PD prescription or clinical management?

Handgrip strength

21. Does your PD unit have access to a handgrip dynamometer?
22. Is handgrip strength routinely measured?
23. If yes, which dynamometer is used?
24. If handgrip strength is not routinely measured, what are the main reasons?

- Lack of time
- Not considered useful
- Patient refusal
- Other (please specify)

Muscle ultrasound

25. Is ultrasound equipment available at your hospital?
26. Does the PD team have experience using ultrasound?
27. Does the PD team have experience specifically in muscle ultrasound assessment?
28. Is muscle ultrasound used routinely for muscle assessment?
29. If yes, how frequently is muscle ultrasound performed?

Bioimpedance

30. Is bioimpedance analysis available in your PD unit?
31. How frequently is bioimpedance performed?
32. Is bioimpedance used to assess muscle mass/body composition in addition to hydration status?
33. Do you use a validated equation to estimate appendicular skeletal muscle mass from bioimpedance measurements?

Section 6. Health-related quality of life

34. Do you routinely assess health-related quality of life (HRQoL) in patients receiving PD?
35. Which instrument(s) do you use?

- KDQOL
- SF-36
- SF-12
- PROMIS
- Other (please specify)

36. When is HRQoL first assessed?
37. Is HRQoL reassessed during follow-up? If yes, at what interval?
38. Does HRQoL assessment influence PD prescription or clinical decision-making?
39. If HRQoL is not routinely assessed, what are the main reasons?

- Lack of time
- Patient refusal
- Not considered useful
- Other (please specify)

Section 6. Digital tools and clinical follow-up

40. Which electronic software/platform is used for clinical follow-up of PD patients?
41. If no dedicated software is used, what are the main reasons?

- No access/software unavailable
- Excessive time required
- Available applications are not considered suitable
- Other (please specify)

42. Which software is used to calculate peritoneal dialysis kinetics?
43. Do you have experience using the GCDP database adapted for this project?

44. Have you experienced any difficulties using this database? If yes, please specify.

Section 7. Perceived Barriers:

45.- If you don’t implementing frailty-informed and comprehensive assessment protocols, why?

- Lack of time
- Absence of specific clinical guidelines
- I do not consider it useful
- Other: please specify.

46.- Have you received formal training in the past 5 years on frailty, sarcopenia, or HRQoL evaluation ¿

47.- Do you agree that frailty influences modality choice and patient prognosis ?

Si/No

48.- Incorporating these assessments would modify their day-to-day clinical workflow ?

Si / No

Supplementary Figure S1.

Study flow diagram

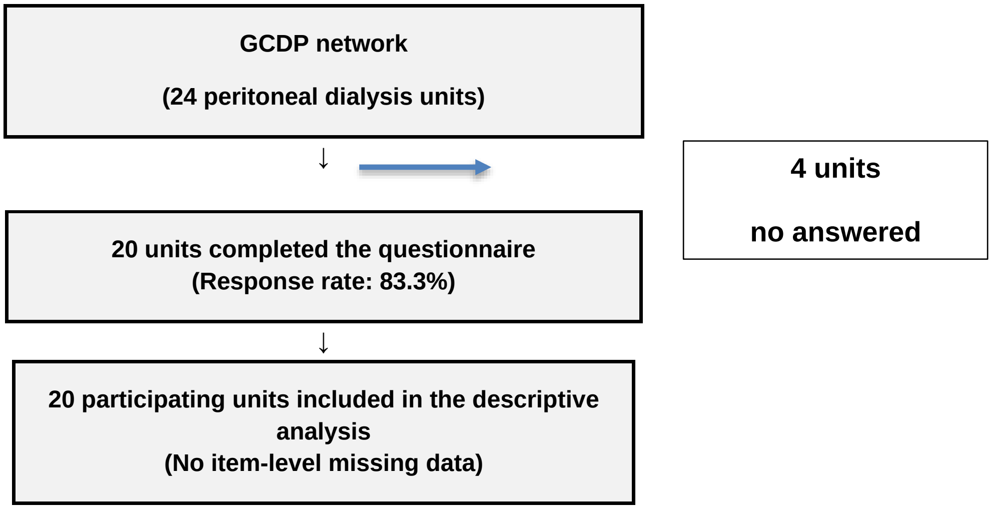

Figure S1. Flow of peritoneal dialysis units through the study. All 24 GCDP units were invited by email; 20 completed the Excel-based questionnaire. All participating units completed all survey items. GCDP, Grupo Centro de Diálisis Peritoneal.
